# Urinary Deposits, Their Diagnosis, Pathology, and Therapeutical Indications

**Published:** 1845-07

**Authors:** 


### ART. IV.

*Urinary Deposits, their Diagnosis, Pathology, and Therapeutical Indications.* By GOLDING BIRD, A.M. M.D. Assistant Physician to and Lecturer on Materia Medica at Guy's Hospital, &c. &c.—*London,* 1844. Small 8vo, pp. 324; with Thirty-one Wood Engravings.

THIS extremely neat and compact little volume contains the valuable lectures published by Dr. Golding Bird in the 'Medical Gazette,' in the early part of 1843, extended and almost completely rewritten; so as to include the latest information on the subject, derived both from the author's large field of public experience, and from foreign sources. "In coming in contact with pupils, in the course of my duties as a teacher of my profession, and in mixing with medical men in practice," Dr. Bird remarks, "I have often found them in want of some work, which would enable them readily to discover the nature of a deposit in the urine, and succinctly point out its pathological and therapeutical indications." The re-introduction of the microscope for the diagnosis of urinary deposits,—which instrument was employed a century ago for this purpose by Van Swieten, —removes the objection which has been frequently urged by practitioners against the possibility of a minute acquaintance with disorders of the urinary secretion; since a minute or two is sufficient for the observer to learn the nature of any variety of sediment, when he possesses such a complete series of types for comparison, as those which are figured in this Manual.

While endeavouring to describe minutely the diagnosis and pathology of urinary deposits, Dr. Bird has not thought it necessary to enter as fully into the consideration of their treatment; this having been already discussed fully by Dr. Prout and Sir B. Brodie. He has made an exception, however, in regard to the oxalate of lime deposit; on which he has enlarged more fully, in consequence of the scanty amount of information as to its pathology and treatment, to be found elsewhere. We shall now briefly follow Dr. Bird through his treatise; with the view of impressing upon our readers our sense of the value of his inquiries, and of making them comprehend the vastly increased facilities which are now presented to them, in the diagnosis of this most important class of diseases. And if there is one set of diseases more than another, in which an accurate *diagnosis* is essential to correct *treatment*, it is that with which we are now concerned. Any mere empirical prescription, applied to a deposit in the urine, whose chemical nature has not been ascertained, either by the microscope or by analysis, is at best but a chance remedy, which is about as likely to be really serviceable, as Morison's pills or Solomon's balm of Gilead. There is no department of medicine, in which pathological science is contributing so much to the improvement of the therapeutic art, as it is doing in this; and there is none in which the improvement may be so readily made available in practice. We regard Dr. Bird's little treatise as possessing very great value in this respect. It is essentially a *practical* one; designed to teach the true interpretation of the class of symptoms presented by abnormal states of the urine; and to lead from these to the pathological conditions of the general system indicated by them. The clearness and precision of thought, which result from the study of natural science, are everywhere apparent in this treatise; and we know of no work which we can more conscientiously recommend to our young authors, as (with the exception of a few inaccuracies of style) a model of correct composition. Unlike too many of those aspirants for literary reputation, whom we have lately had occasion to chastise with unsparing hand, Dr. Bird has, in the first place, something of real novelty and importance to tell his readers; and, in the second, he has told it simply, briefly, and well. His first paragraph will show that he is far from resting in the empirical application of remedies to the various morbid phenomena, which it is his object to render easily distinguishable; but that he estimates them at their true value,—namely, as *signs* of disease, not in themselves *diseases.*

"In availing himself of the phenomena presented by the urine in disease, it is essential that the practitioner should not fall into the error of regarding a knowledge of the morbid condition of the secretion as alone essential in directing his treatment; nor must he commit the equally serious mistake of regarding every deviation from the natural conditions of the urine as constituting a disease *per se.* The only view that can be legitimately taken of such conditions is to regard them not as constituting entities of morbid action, but as one of a series of pathological changes going on in the system, and more valuable than others as an index of disease, in consequence of the facility with which it is detected. Hence every abnormal state of the secretion in question should be regarded rather as an indication of some particular phase of morbid action, than as constituting the ailment itself." (p. 2.)

On the first chapter, which treats of the *Physiological Origin and Physical Properties of the Urine,* we need make little remark, since Dr. Bird's views in the former points closely correspond with those which we have recently had several occasions of expressing; and on the latter part of the subject, there is, from its very nature, little new to be said. We must not omit to notice, however, a very useful table of the different colours presented by the urine in disease; showing the usual causes of these colours, the means of determining them by chemical reagents, and the pathological conditions which they indicate. The following observation we believe to be novel. It is well known that the urine occasionally coagulates spontaneously on *cooling*; but in a few rare instances, occurring chiefly in urine loaded with oxalate of lime, Dr. Bird has found it quite fluid when cold, and gelatinizing when *heated,* retaining its transparency. At the end of this chapter is a short account of the mode in which polarized light has been recently applied by M. Bouchardat (according to the plan originally proposed by M. Biot) to the detection of sugar in urine; but Dr. Bird is not of opinion that it can ever be generally employed, on account of the many difficulties in the way of its practical application.

The second chapter embraces the *chemical physiology of the urine;* and we shall notice a few of the chief points of interest discussed in it. Notwithstanding the doctrine recently propounded by Liebig, that the uric acid of the urine is held in solution by the phosphate of soda, combining with a part of the base, and setting free a portion of the phosphoric acid, Dr. G. Bird still adheres to the opinion of Dr. Prout, that the uric acid is combined with ammonia; and he states it as a conclusive argument in favour of this view, that, if healthy urine be slowly evaporated in an air-pump vacuum, it soon becomes turbid from the formation of clouds of urate of ammonia,—as also occurs when urine of rather high specific gravity is exposed to cold. The objection urged by Becquerel,—that a single drop of nitric acid is sufficient to precipate all the uric acid naturally contained in a considerable quantity of urine,—is of no weight when it is remembered that this quantity is quite sufficient to neutralize *all* the ammonia that was previously in combination with the uric acid. Hence a modification is required in Liebig's views; since, although they account for the solution of uric acid in warm urine, for the deposition of crystals of impure uric acid on cooling, and for the natural acidity of the fluid, they do not account for the deposits of urate of ammonia just noticed. These are believed by Dr. Bird to result from the action of nitric acid on the triple phosphate of soda and ammonia; which salt, or its elements, may be regarded as a constant constituent of healthy urine. When uric acid is mixed with a warm solution of this triple phosphate, urate of ammonia is formed, and is deposited in a crystalline form on cooling; and phosphoric acid is evolved,—either free, or in combination with a base forming an acid salt. The following view is proposed by Dr. Bird, as to the mode in which uric acid exists in urine.

"Uric acid, at the moment of separation from the blood, meets the double phosphate of soda and ammonia derived from the food, and forms urate of ammonia evolving phosphoric acid, which thus produces the natural acid reaction of the urine. If the whole bulk of the urine be to the urate of ammonia formed not less than about 2700 to 1, the secretion will, at the ordinary temperature of the air, remain clear; but if the bulk of the fluid be less, an amorphous deposit of the urate will occur. On the other hand, if an excess of uric acid be separated by the kidneys, it will act on the phosphate of soda of the double salt; and hence, on cooling, the urine will deposit a crystalline sediment of uric acid sand, very probably mixed with amorphous urate of ammonia, the latter usually forming a layer above the crystals, which always sink to the bottom of the vessel." (p. 42.)

We cannot but regard this view as highly probable; and think that in this, as in other instances, an insufficient acquaintance with the phenomena of health and disease has been the cause of error in the determinations of Liebig. The idea suggested by him, however, in regard to the part performed by the phosphates in the composition and reactions of urine, is obviously of great value; and the chief modification proposed by Dr. Bird consists in the substitution of the triple phosphate of soda and ammonia, for the simple bibasic phosphate of ammonia, so as to account for the deposition of urate of ammonia, as well as of uric acid.

In his estimate of the views of Liebig, in regard to the sources of the production of urea and uric acid, Dr Bird's opinions completely accord with those heretofore expressed by ourselves; and they are borne out in a very striking manner by the recent experiments of Lehmann, who has shown that the quantity of *urea* contained in the urine, during the use of an exclusively animal diet, was between three and four times as much as that, which was present during a diet of non-azotized food, and which might therefore be regarded as representing the actual *waste* of the system. The proportion of *uric acid* was twice as great in the former case as in the latter.

In regard to the supposed presence of lactic acid or lactate of ammonia in the urine, Dr. Bird seems to rely upon the recent experiments of Liebig as proving that chemists have been in error in this matter; and that what has been mistaken for lactic acid is really a peculiar crystallizable matter not hitherto described. But considering the close relation which lactic acid bears to some of the most ordinary elements of food, he seems to regard its occasional presence as not unlikely, even though it be not one of the normal constituents of urine.

Dr. Bird's researches accord with those of Liebig, in establishing the presence of hippuric acid as a normal component of urine; but he thinks that its quantity in health is not constant, and that it is always,—except after the ingestion of benzoic or cinnamic acid,—much less than has been stated. The following idea appears to us as probable as it is ingenious:

"It is possible that hippuric acid may constitute a means by which carbon may be evolved from the system by the kidneys; and it is probable that, in cases in which the proper emunctories of this substance, the lungs and liver, are deficient in their function, the kidneys may partially compensate for this, by secreting a larger proportion of hippuric acid. It is remarkable that this substance, next to the bile, is the richest in carbon of any of the products of vital chemistry; and hence it very probably performs an office of great importance in the body. A comparison of the per-centage composition of the organic material of human bile, from the analysis of Dr. Kemp, with that of hippuric acid, will show the relation between them, *quoad* the amount of carbon:

| Human Bile. | Hippuric Acid. |
|---|---|
| 68·40 | 63·93 |
| 3·44 | 8·21 |
| 10·13 | 4·64 |
| 18·03 | 23·22 |
| 100·0 | 100·0" (p. 51) |

This idea corresponds very well with the fact, that the ingestion of a large quantity of carbonaceous food is as favorable to the production of hippuric acid in man, as in the herbivorous animals; thus Dr. Bird learned from Professor Liebig, that a girl in the hospital at Wurzburg having refused all food excepting apples (of which she devoured an enormous quantity), her urine was found to contain a large quantity of hippuric acid, but no uric,—like the urine of a horse or cow.

On the nature of the red colouring matter of the urine, Dr. Bird is at issue with the other authorities on the subject. By Dr. Prout it is regarded as a purpurate of ammonia; and this by Liebig has been termed murexid. Dr. Bird adduces very sufficient reasons, however, for the belief, that the colouring matter in question is a substance *sui generis*, and is not identical with either. Thus *purpurine* (as he terms it) is readily soluble in alcohol; which menstruum is without action on purpurate of ammonia or murexid; and the reactions of the two with various tests are very different.

On the *chemical pathology of uric acid and its combinations*, which forms the subject of the third chapter, we may be very brief, as this topic has frequently engaged our attention of late. The chief feature in Dr. Bird's treatment of the subject, consists in the fulness of his account of the microscopic characters of the deposits; which is elucidated by numerous woodcuts, enabling any person possessed of a tolerable microscope to recognize them without any difficulty. The figs. 6, 7, (p. 68,) represent two varieties which are sometimes met with in the crystallization of uric acid, when it forms coarse sand of a deep orange or red hue. The little aggregations of crystals here figured may be regarded, indeed, as minute calculi; and those of the lozenge form, which are particularly met with where a marked tendency to the formation of calculi exists, are not unfrequently found crystallized upon a hair, like sugar-candy on a thread.

Dr. Bird lays much stress, and we think quite deservedly, on the *skin* as a channel for the excretion of azotized matter, and attributes the increased amount of urate of ammonia found in the urine after a check to perspiration, to the temporary suspension of that excretion, and the compensating action of the kidneys. In proof the excretion of azotized matter from the skin,—which has been doubted and even denied by some, he mentions the followiong interesting experiment: "Dr. Faraday calcined pure river sand, and on heating it with hydrate of potass, it yielded no trace of ammonia. On merely passing this sand over his hand, and then treating it in a similar manner, ammonia evolved. A piece of ignited asbestos by mere pressure for a short time between the fingers, absorbed enough of some nitrogenized organic matter, to evolve ammonia, when heated with hydrated potass." (p. 77.)

After discussing the theory of Liebig in regard to the causes of excess of uric acid,—on which we find Dr. Bird's views quite in accordance with our own,—he thus continues:

"Excluding all abstract theories, whenever an excess of uric acid or its combinations with bases occurs in the urine, a normal quantity of water being present (30 or 40 ounces in twenty-four hours), it may safely be inferred that one or other of the following states exists."

| | |
|---|---|
| A. Waste of tissue more rapid than the supply of nitrogenized nourishment, as in . . | Fever, acute inflammation, rheumatic inflammation, phthisis. |
| B. Supply of nitrogen in the food greater than is required for the reparation and supply of tissue, as in . . . . | Excessive indulgence in animal food, or the quantity of food remaining the same, with too little bodily exercise. |
| C. Supply of nitrogenized food not being in excess, but the digestive functions unable to assimilate it . . . . | All the grades of dyspepsia. |
| D. The cutaneous outlet for nitrogenized excreta being obstructed, the kidney is called upon to compensate for this deficient function . | All or most stages of diseases attended with arrest of perspiration. |
| E. Congestions of the kidneys, produced by local causes . . . | Blows and strains of the loins, diseases of the genital apparatus, &c. |

The following is, we believe, a novel suggestion in regard to the treatment of the uric acid diathesis.

"The remarkable solvent action of phosphate of soda on uric acid, to which Liebig has lately directed attention, inspires a hope that its administration may be of use in cases of calculous disease, by impregnating the urine with an active solvent. All that is required to ensure this drug reaching the urine is to administer it in solution sufficiently diluted; ℈j to ʒss might be administered in any vehicle, as in broth or gruel, as when diluted the phosphate tastes like common salt, and few persons object to its flavour. I have administered this drug in two very chronic cases of uric acid gravel; and in one with the effect of rapidly causing the disappearance of the deposit. This occurred in the person of a lady about forty years of age; who had, at my wish, for some weeks used the artificial Vichy water of the German Spa at Brighton without relief. The triple salt, ammonio-phosphate of soda, would perhaps be a more active remedy than the simple phosphate; but its disagreeable flavour constitutes one objection to its employment." (p. 98.)

The following we consider a very just appreciation of the value of different classes of remedies, in the treatment of these disorders.

"It is important to bear in mind that, by the employment of remedies capable of dissolving a deposit in the urine, we are merely palliating, not curing, the disease. And we must never lose sight of the great importance of endeavouring to remove that pathological state of the whole system, or of any particular organ, which may be the exciting cause of the calculous formation. Nothing but a careful investigation of symptoms can put us in possession of the knowledge necessary for this purpose; still, solvent remedies are not to be despised; for when the disease is chronic, and does not readily yield to treatment, it is of the utmost importance to prevent the formation of a calculus, or to lessen the irritation produced by the presence of gravel, whilst endeavouring to remove the primary affection which led to the formation of the deposit." (p. 101.)

Dr. Bird's observations upon Purpurine, the chemical pathology of which is discussed in the fifth chapter, possess considerable importance; on account of the serious lesions of which he considers its presence to be frequently indicative. This substance is usually found in combination with urate of ammonia; which has so strong affinity for it, as always to become deeply coloured by purpurine, when deposited from urine in which it is present.

"The presence of an excess of purpurine is almost invariably connected with some functional or organic mischief of the liver, spleen, or some other organ connected with the portal circulation. The appearance of a flesh-coloured deposit in the urine is the commonest accompaniment of even slight derangement of the hepatic function, as every case of dyspepsia occurring in gin-drinkers points out. The intensity of colour of the deposit appears to be nearly in relation with the magnitude of the existing disease. In the malignantly-diseased, in the contracted, hobnail, or cirrhosed liver, the pink deposits are almost constantly present in the urine. They also are of frequent occurrence in the hypertrophy of the spleen following ague. The most beautifully coloured deposits I have seen occurred in ascites connected with organic disease of the liver; and I think I have received some assistance in the diagnosis between dropsy depending upon hepatic and peritoneal disease, in the presence of pink deposits in the former, and their general absence in the latter. I have occasionally seen the deposits in question occur in phthisis; when large quantities of pus were poured out from vomicæ; as well as in deep-seated suppuration, as in psoas abscess. But even in these cases, the portal circulation is probably more or less influenced. My experience, indeed, leads me to express a firm belief, that an excess of purpurine is almost pathognomonic of disease in the organs in which portal blood circulates." (p. 110.)

The succeeding chapter, on the *chemical pathology of cystine,* contains, we believe, a fuller account of that curious substance, than is elsewhere to be met with; but as the rarity of its occurrence, and our want of therapeutic indications regarding its treatment, render it a subject of little practical importance, we shall dismiss it with a very brief notice. Cystine is considered by Dr. Bird as a derivative of albumen, or of tissues into which it enters; and appears to be the result of derangement of the secondary assimilative processes, essentially connected with the elimination of sulphur, every ounce of cystine containing more than two drachms of this element. There appears sufficient evidence that its appearance is connected with a scrofulous diathesis, and that it is disposed to be hereditary. Dr. Bird agrees with Becquerel in referring cystine to the same origin with urea and uric acid; and gives a formula, which shows that 1 urea + 1 uric acid + 4 sulphuretted hydrogen, will be equivalent to 2 cystine + 4 nitrogen.

To Dr. Bird's account of the *pathology of oxalate of lime,* which forms chapter vii, we are especially desirous of directing the attention of our readers; since he has proved, quite satisfactorily in our opinion, that this substance is a far more frequent deposit than has been hitherto supposed. It is remarkable that even the most recent writers on calculous affections, whilst tracing the history of oxalate of lime as a material of calculous formations, should not have detected this substance in urinary deposits. Thus in Dr. Prout's classical work, the remarks made on the oxalate acid diathesis apply to the cases in which the oxalate of lime has existed in a truly calculous form; or to those in which the presence of oxalic acid is rather suspected than proved; and the only case of the spontaneous occurrence of oxalate of lime in the urine, quoted either by M. Rayer or by Dr. Willis, is one which some years ago occurred to Dr. Bird himself, and was described by Dr. Brett. The chief results of Dr. Bird's subsequent inquiries have been already given to the world in the Medical Gazette; but we shall not apologise to our readers for here giving a brief analysis of them, since they cannot be too strongly impressed upon their minds.

Dr. Bird's examination by the microscope, and by chemical analysis, of many hundreds of specimens of diseased urine, has led him to discover the *comparative frequency* of oxalate of lime in the urine, in fine and well defined octohedral crystals; and to establish the connexion between the occurrence of this substance, and the existence of certain definite ailments, all characterized by great nervous irritability. Dr. Bird's field of observation having been restricted to one set of conditions, he cannot, of course, form an opinion of the degree of connexion, which he seems to suspect, between the formation of this salt, and the depressing influences always more or less active in large and densely-populated cities; but he has no hesitation in declaring it to be the result of his own experience, that, in the metropolis, the *oxalate is of far more frequent occurrence in the urine than the deposits of earthy phosphates.* The cause of its having been so generally overlooked appears to lie in the curious fact, that this salt never subsides so as to form a distinct deposit, but remains for days diffused through the fluid; even when present in so large a quantity, that each drop of the urine, when placed under the microscope, is found loaded with the crystals. Moreover, as its refractive power nearly approaches that of the urine, its presence occasions little or no turbidity, and the floating particles escape even attentive observation by the unaided eye. Dr. Bird's figures, figs. 17, 18, 19, represent the forms exhibited by these crystals under the microscope. The first shows their usual aspect,—that of beautifully-formed transparent octohedra, with sharply-defined edges and angles. When allowed to dry upon glass, and then examined, each crystal presents a very curious appearance,—resembling two concentric cubes, with the angles and sides opposed, the inner one transparent, the outer black; so that each resembles a translucent cube set in a black frame, as shown in the second figure. And in a very few cases, the oxalate is met with in very remarkable crystals, or rather masses of crystals, shaped like two kidneys with their concavities interposed, and sometimes so closely approximating, as to appear circular, the surfaces being finely striated. This zeolitic crystallization was found mixed with, and ultimately replaced by, those of the ordinary octohedral variety. In almost every case of oxalic urine, a very large quantity of epithelial scales was found; and the white deposit of these frequently served to indicate the probable presence of the oxalate; whilst the freedom of oxalic urine from such admixture, formed the exception to the general rule. The following are Dr. Bird's conclusions in regard to the other characters of oxalic urine.

"1. That in rather more than one third of the cases of oxalic urine, uric acid or urates existed in large excess, forming the great bulk of the existing deposit.

"2. That in all, there exists a greater proportion of urea, than in natural and healthy urine of the same density; and in nearly 30 per cent. of the cases, so large a quantity of urea was present, that the fluid crystallized into nearly a solid mass on the addition of nitric acid.

"3. That the urate of ammonia found in the deposits of oxalic urine is occasionally tinted of a pink hue.

"4. That an excess of phosphates frequently accompanies the oxalate.

"5. That no evidence of free sugar has occurred in the specimens I have examined." (p. 135.)

Although the close chemical relation subsisting between oxalic acid and sugar naturally leads to the belief that a similar relation exists between diabetic and oxalic urine, yet Dr. Bird has not been able to detect any such relation; but he has, on the contrary, sought in vain for oxalate of lime in diabetic urine, or for sugar in oxalic urine. On the other hand, finding an excess of urea and uric acid in a large proportion of specimens of oxalic urine, he thinks it a legitimate conclusion that this disease is in reality a form of azoturia,—oxalic acid having been shown, by Liebig and Wohler, to be readily derivable from uric. We are not required, however, by this supposition, to refer the origin of the oxalic acid to the metamorphosis of the tissues; for, on the other hand, several circumstances lead to the belief, that, like urea and uric acid when in excess, it is to be referred to a *primary* mal-assimilation or non-assimilation of the elements of food. Thus the quantity of the oxalate is always greatest after a full meal; and its amount is much less in the *urina sanguinis,* or that passed on rising in the morning, from which it may be even altogether absent. Moreover it diminishes under the influence of a carefully-regulated diet; and increases again on a return to unwholesome food,—some articles which are themselves quite free from oxalic acid, having the power of at once causing the excretion of this substance in large quantities. Since, however, it has been proved by the recent inquiries of Dr. Buchanan, that sugar may be detected in the serum of even healthy blood, drawn soon after the ingestion of food containing a large proportion of saccharine or farinaceous matter,—a fact to which Dr. Bird does not here allude,—we cannot feel quite satisfied as to the entire absence of the relation between a mal-assimilation of the saccharine principle, and the appearance of oxalic acid in the urine. It is a confirmation of Dr. Bird's view, however, that an increase in the deposit of uric acid is generally (according to his experience) the first effect of remedies which diminish the amount of oxalate. The following are the general symptoms which he has observed to accompany this diathesis:

"Persons affected with the disease under consideration are generally remarkably depressed in spirits, and their melancholy aspect has often enabled me to suspect the presence of oxalic acid in the urine. They are generally much emaciated, excepting in slight cases, extremely nervous, and painfully susceptible to external impressions, often hypochondriacal in an extreme degree, and in the majority of cases labour under the impression that they are about to fall victims to consumption. They complain bitterly of incapability of exerting themselves; the slightest exertion bringing on fatigue. In temper they are irritable and excitable; and in men the sexual power is generally deficient, and often absent. A severe and constant pain, or sense of weight across the loins, is generally a prominent symptom. The mental faculties are generally but slightly affected, loss of memory being sometimes more or less present. Well-marked dyspeptic feelings are always complained of. Indeed, in most of the cases in which I have been consulted, I have been generally told that the patient was ailing, losing flesh, health, and spirits, daily; or remaining persistently ill and weak, without any definite or demonstrable cause. In a few the patients have been suspected to be phthisical. It is, however, remarkable that I have yet met with very few cases in which phthisis was present." (p. 141.)

In regard to the exciting cause of the secretion of oxalic acid, Dr. Bird states that they were, in the majority of cases at least, generally well marked; and that in almost all, the predisposing cause was nearly the same; viz., a chronic and persistent derangement of the general health, or the lowering of the system by previous acute disease. The exciting cause has generally consisted in some circumstance, which has determined the irritation to the urinary organs,—such as exposure of the lower part of the spine to cold, mechanical violence over the region of the kidneys, unnatural excitement of the genital organs, &c.; but, in many instances, no other obvious cause existed than great and protracted mental effort. The treatment, in the majority of cases, was very successful; chiefly consisting in minute attention to the general health, and especially to the due performance of the digestive operations, and to the cutaneous secretion. Dr. Bird speaks highly of the effects of colchicum; the use of which increases the normal quantity of uric acid in the urine. In two cases, in which the oxalate of lime existed before its employment, uric acid appeared after a few days as a deposit, and almost entirely replaced the oxalate.

As an appendix to this chapter, Dr. Bird gives seven illustrative cases, selected rather as presenting the chief varieties of ailment connected with the oxalic diathesis, than for the sake of pointing out the treatment. These will be read with much interest; and will doubtless serve to direct more general attention to this important class of disorders. Some interesting researches by Dr. Aldridge, of Dublin, on the products of the decomposition of uric acid, are also added; from which it appears that by simple chemical processes, not only the oxalate and carbonate of ammonia, but also the formic and hydrocyanic acids, may be generated from it. Hence it appears probable that as very slight variations in the temperature, degree of concentration, &c. of the urine, when it is boiled, will affect its products, analogous variations may do the same within the body; and that, under the influence of morbid conditions, hydrocyanic acid and other compounds of cyanogen may thus be produced. Indeed one case is on record, in which hydrocyanic acid was detected in the urine; and there are several, in which ferro-cyanic acid and per-cyanide of iron have been discovered. The development of *known* poisons in the body under the influence of disease, merits the utmost attention, from its great pathological importance; but it would be satisfactory to have the possibility of the production of hydrocyanic acid in the system more clearly established; since Brugnatelli's case just alluded to can scarcely be regarded as alone sufficient for the proof of so important a position; and it would be requisite to watch very closely for the exclusion of all possible sources of error, in regard to the materials of the *ingesta,* before any inferences should be founded upon the appearance of the acid in the urine.

The *chemical pathology of the earthy salts,* forming the subject of the next chapter, is treated of with similar fulness and precision; but our limits forbid our enlarging upon the several interesting questions which the discussion of this topic involves. The microscopic characters of the earthy phosphates are extremely precise; so that they may be at once recognized (with the exception of phosphate of lime, which is amorphous,) by this kind of examination. Of the circumstances under which these deposits are prone to occur, Dr. Bird gives the following account:

"The occurrence of deposits of the earthy phosphates in the urine must be regarded as of serious importance, always indicating the existence of important functional, and too frequently even of organic, mischief. One general law appears to govern the pathological development of these deposits, viz., that they always exist simultaneously with a depressed state of nervous energy, often general, rarely more local, in its seat. Of the former, the result of wear and tear of body and mind in old people, and of the latter the effects of local injury to the spine, will serve as examples. It is true that, in the majority of these cases, there is much irritability present, there is often an excited pulse, a tongue white on the surface and red at the margin and tip, with a dry, often imperspirable, occasionally hot skin. Still, it is irritability with depression, a kind of erythism of the nervous system, if the expression be permitted, like that observed after considerable losses of blood." (p. 176.)

Similar observations, long since made by Dr. Prout, have appeared to us to indicate (when taken in connexion with other phenomena) that the presence of phosphates in the urine, in any unusual amount, is ordinarily due to an excessive *waste* or metamorphosis of nervous tissue, resulting from undue functional activity. It is well known that phosphorus enters into the composition of this tissue, more largely than into that of any other of the soft tissues of the body; and this will, of course, be set free, and will present itself in the excretions, whenever disintegration of the nervous tissue takes place. Now that this disintegration is proportional to the functional activity of the tissue, seems probable, not on general grounds alone, but from various phenomena connected with its nutrition, on which we cannot now stop to dwell. The following case related by Dr. Bird is one of the most satisfactory proofs we have ever met with, of the connexion we have alluded to. It is by him adduced, as one out of many similar illustrations, of the dependence of phosphatic deposits upon the depressed but irritable state of the system, resulting from over-exertion of mind or body. In our view, both are to be referred to a common cause,—the previous *waste* of nervous matter, which will produce the general depression of nervous power that is marked in these cases, as well as the phosphatic deposit in the urine.

"The simplest examples of this kind that have occurred to me have been in the cases of individuals of nervous temperament, who have periodical duties to perform, requiring extreme mental tension and bodily exertion. I have witnessed this state of things several times in clergymen, especially in those who, from the nature of their secular engagements, have been compelled to lead sedentary lives during the week, and to perform full duties on Sundays. The best illustration of this I ever met with, was in the person of a well-known and deservedly popular clergyman; who, from his connexion with a public school, scarcely used any exercise during the week, whilst on Sunday he performed duty thrice in his church. This gentleman was a tall thin person, of dark complexion, lustrous eyes, and almost phthisical aspect. He was the subject of constant dyspepsia. The urine passed on Saturday evening, as well as on the Sunday morning, although repeatedly examined, was healthy, except in depositing crystals of urate of ammonia, and being of a high specific gravity. Before his Sunday duties were completed, he almost invariably became the subject of extreme fatigue, with a painful aching sensation across the loins, in addition to the flatulence and epigastric uneasiness under which he always laboured. The urine voided before retiring to rest after the severe exertions of the day, was almost constantly of a deep amber hue, high specific gravity, and deposited the triple phosphate in abundance. The urine of Monday would contain less of this salt, which generally disappeared on the following day, and once more reappeared on the following Sunday evening. I had an opportunity of observing this state of things for several weeks; and it ultimately disappeared by the patient relaxing from his duties, and enjoying the amusement of travelling for a few weeks." (p. 179.)

The view we have taken of the immediate cause of the deposition of the phosphates, is borne out by the statement of Dr. Prout, which is fully confirmed by Dr. Bird, as to the great value of narcotics in the treatment of this diathesis. When, in consequence of over-exertion, or undue irritability of the nervous system, its *waste* is too rapid, and there is need of the restoration of its powers by the quiet exercise of the nutritive process, there can be nothing so favorable to this recovery as the administration of narcotics, which keep down its functional activity.

Our assigned limits prevent us from following Dr. Bird through the remaining portion of his account of urinary deposits; in which the less frequent appearances are noticed, and the mode of determining their characters described. We may stop, however, to remark, that Dr. Bird continues well satisfied of the accuracy of what he formerly published on the subject of *kiestein*, the peculiar ingredient which appears to be pretty constantly present in the urine of pregnant women. The greasy aspect of the pellicle formed by this substance seems due not so much to the presence of fat, as to the glistening of numerous crystals of triple phosphate. He admits, however, that some fat is present; the experiments of Lehmann having proved its existence. It is further interesting, that this fatty matter closely resembles butter, and is convertible into butyric acid. Dr. Bird gives two interesting cases, in which the caseous pellicle was formed on the urine of women almost immediately on their ceasing to suckle,—one of these individuals being pregnant, and the other not. These facts seem to give strong corroboration to the belief, that the urinary excretion furnishes the means of getting rid of the materials of the mammary secretion, which are in preparation *before* they are wanted, and which remain *after* the call for them has ceased. Whether these materials are being prepared in the blood, and are separated by the kidneys when the mammary gland does not act, or whether they are first separated (as Dr. Bird seems to suppose) by the mammary gland, and are then reabsorbed to be eliminated by the kidneys, is a question which we have as yet no means of determining.

We shall close our review of this valuable work with a brief notice of its last chapter, entitled "Remarks on the Therapeutical Employment of Remedies influencing the functions of the Kidneys;" in which an attempt is made to account in some degree for the capricious action of these remedies, and to lay down rules for the better guidance of the practitioner in administering them. Adopting the view, which is we believe the one best in accordance with sound physiology,—that soluble substances find their way into the circulating system, not through the lacteals, but *directly* by means of the blood-vessels of the villi,—he lays great stress on the mode in which salines should be administered; adopting Liebig's view, that when a saline solution of *lower* specific gravity than the blood is introduced into the stomach, it will be absorbed by endosmose, and will produce diuresis; whilst the ingestion of a *strong* solution will cause an exosmose of the fluid parts of the blood into the intestines, and will produce watery purging. Hence Dr. Bird lays down the following rule: "1. Whenever it is desirable to impregnate the urine with a salt, or to excite diuresis by a saline combination, it must be exhibited in solution, so diluted as to contain less than five per cent. of the remedy, or not more than about 25 grains in an ordinary draught. The absorption of the drug into the capillaries will be ensured by a copious draught of water or any diluent, immediately afterwards." Again, the absorption of salines will be impeded by any cause which produces congestion of the mesenteric veins; and if that cause be obstruction in the portal circulation, there will be not only deficient absorption, but a retardation of the action of what has found its way in, upon the kidneys. Hence, "2. When the urine contains purpurine, or other evidence of portal obstruction exists, the diuretics or other remedies employed should be preceded or accompanied by the administration of mild mercurials,—taraxacum, hydrochlorate of ammonia, or other cholitic remedies. By these means, or by local depletion, the portal vessels will be unloaded, and a free passage obtained to the general circulation. 3. In cases of valvular or other obstructions existing in the heart and large vessels, it is next to useless to endeavour to excite diuretic action, or to appeal to the kidneys by remedies intended to be excreted by them. The best diuretics here will be found in whatever tends to diminish the congested state of the vascular system. and to moderate the action of the heart; as digitalis, colchicum, and other sedatives, with mild mercurials." (p. 293.) In the justice of these views we fully coincide; and we doubt not that by careful attention to them the practitioner will find many of his difficulties diminished, if not altogether removed. Indeed we believe we might say, that the rules in question are nothing but the expression of the most satisfactory experience on the subject; but when their *rationale* is determined, they lose their merely *empirical* character, and attain the rank of *scientific principles*, whose application is, in judicious hands, far more extensive and certain.

In conclusion, we take leave of Dr. Bird's work, as we began, with a strong recommendation to our readers to possess themselves of it, and to make themselves practically acquainted with its contents; satisfied that they cannot do so, without benefit to themselves and their patients. And we trust that Dr. Bird will persevere in the line of research which he has chosen; since we are confident that he is peculiarly fitted to elucidate its difficulties, and to extend those applications of science to practice, in which the present time is peculiarly fertile.

